# Integrins in cancer: insights into mechanisms and therapeutic potential

**DOI:** 10.1186/s12964-026-02828-w

**Published:** 2026-03-27

**Authors:** Parth Agarwal, Rachana Raman, Prasoon Agarwal, Vijendra Prabhu, Praveen Kumar

**Affiliations:** 1 Manipal Institute of Technology, Manipal Academy of Higher Education, Manipal, India; 2 National Bioinformatics Infrastructure Sweden (NBIS), SciLifeLab, Division of Occupational and Environmental Medicine, Department of Laboratory Medicine, Lund University, Lund, 22362 Sweden

**Keywords:** Cancer stem cells, Integrins, Tumor microenvironment, Plasticity, Therapeutic targeting, Extracellular matrix

## Abstract

Integrins are transmembrane glycoproteins that act as essential adhesion receptors, allowing cells to communicate with the extracellular matrix (ECM). This interaction not only helps cells regulate adhesion, but also transmits signals that guide a variety of cellular processes. Once bound to the ECM, integrins play an important role in the cell differentiation, migration, proliferation, and survival, thereby maintaining tissue homeostasis. However, when integrin signaling becomes dysregulated, it is often associated with tumor development and progression. Abnormal integrin activity promotes uncontrolled cell growth, resistance to apoptosis, and the promotion of angiogenesis in tumors. They also provide resistance to therapies by disrupting growth suppressors. Due to their documented role in the process of tumorigenesis, integrins have become an interesting target for anticancer therapy. In this review, we discuss the function and structure of integrins, emphasizing how altered signaling in integrins consequently leads to cancer formation, progression, and metastasis. In addition, we review existing therapies that target integrins, discuss their limitations, and look at the future of integrin-based therapies. This review deepens our understanding of integrins, their role in cancer, and their possible role as a cancer biomarker.

## Introduction

Integrins are found across a wide range of organisms, including mammals, zebrafish, chickens, and even lower eukaryotes, such as sponges, the fruit fly *Drosophila melanogaster,* and the nematode *Caenorhabditis elegans* [1]. Structurally, integrins are heterodimeric proteins composed of α and β subunits that are primarily localized to the plasma membrane, where they function as major receptors for ECM components [2]. Through their ability to mediate cell–ECM adhesion, integrins play a central role in regulating essential cellular processes, including migration, survival, and proliferation [3]. A defining feature of integrins is their capacity for bidirectional signaling, which allows for both internal–external and external-internal signaling [4]. By binding to ECM ligands, integrins provide the required adhesion for both cell invasion and motility, while also influencing ECM remodeling where they control the activity and localization of proteases [5]. Beyond their role in cell adhesion and ECM remodeling, integrins are important in the immune system. For example, intracellular adhesion molecules (ICAMs), are expressed on antigen-presenting cells (APCs) and the inflamed endothelium, and help guide immune responses. Numerous studies have also linked changes in integrin activity to tumorigenesis. Altered integrin activity is commonly associated with tumor development and progression, indicated by an increase in integrin signaling and altered adhesion [4]. Integrin signaling controls multiple functions in cancer cells, including their migration, survival, proliferation, and invasion. Integrins are also commonly expressed in cells associated with tumors, and can strongly influence the malignancy of the tumor [5]. Various cells make up the tumor microenvironment (TME); this includes endothelial cells, cancer-associated fibroblasts, immune cells such as microglia, tumor-associated macrophages, cancer stem cells (CSCs), and more. Due to their bidirectional nature, integrins regulate cell–cell communication amongst TME cells that activate tumor-supporting functions [6]. It was observed that certain integrin subunits act as surface markers for CSCs in certain cancers, such as colorectal, prostate, breast, and squamous cell carcinomas [7]. A recent study found that integrins are present in lung cancer as a CSC marker, where they promoted the self-renewal of CSCs along with tumor spread and therapy resistance [8]. These behaviors are closely linked to phenotypic plasticity, a hallmark of cancer that enables tumor cells to dynamically transition across distinct phenotypic states, including rans-differentiation, de-differentiation, and blocked de-differentiation [9]. Within the TME, integrins present in immune, endothelial, and peritumoral fibroblast cells are a key factor in the transdifferentiation of these cells [3]. Due to the active role of integrins in tumor development, they have become a desired target in anticancer therapy. Current integrin-targeting therapies that are under clinical trials include arginine-glycine-aspartic acid (RGD) peptide memetics, and monoclonal antibodies (mAbs) [10]. Nanotherapeutics have also been seen as a favored approach due to their multifunctional potential, along with gene therapy, small molecule therapy, photothermal therapy, integrin-based gene delivery, and nanomaterials that target integrins [11].

## Integrins

### Structure

**Fig. 1:**
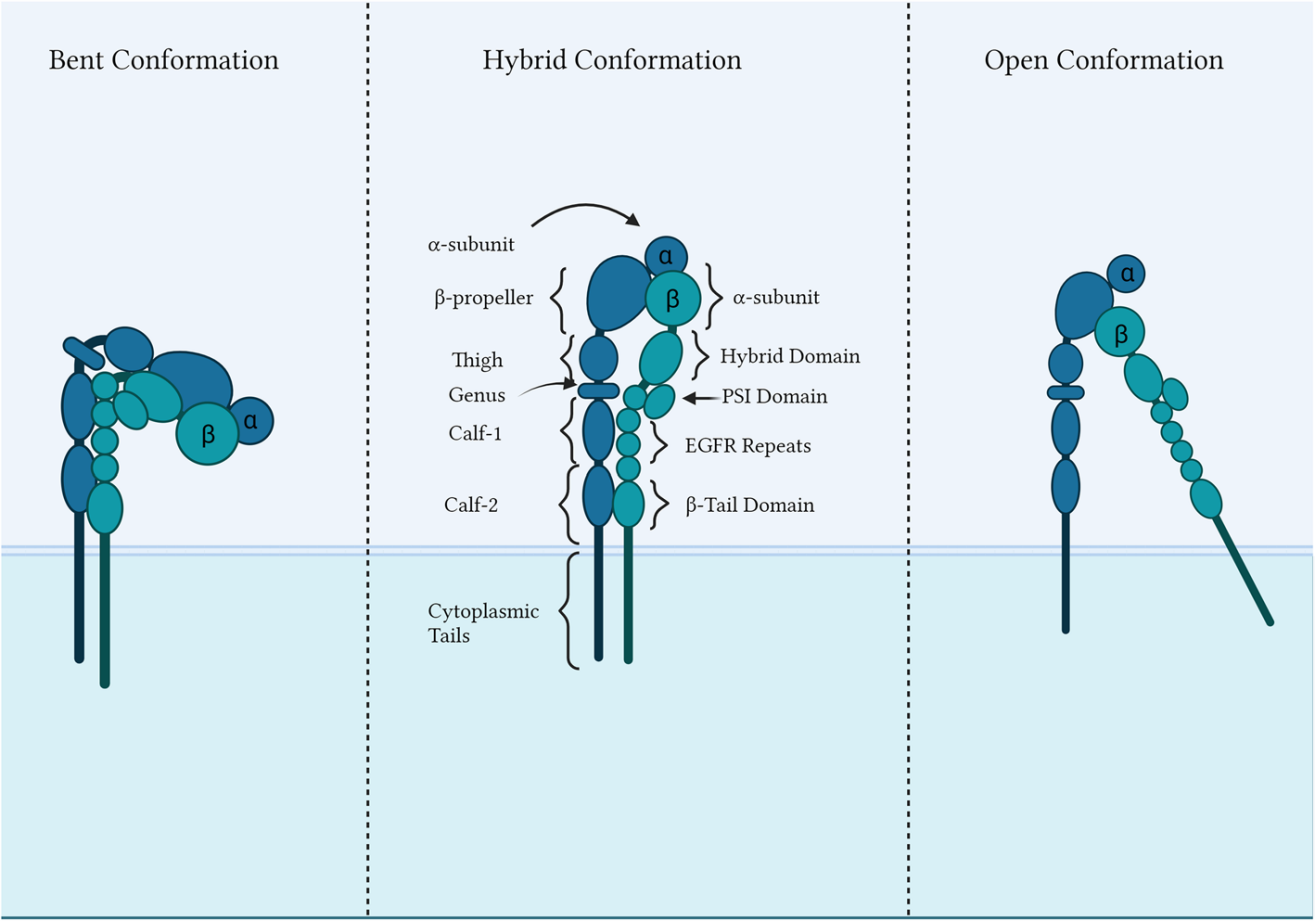
Structure of integrins along with their different conformations (Created with Biorender.com) The conformation of an integrin is largely responsible for its function, and the orientation of each composite unit relative to the others defines the nature of the conformation. The three possible conformations are termed as bent, hybrid and open, which are visualized in Figure 1 (L-R, respectively). Changes in conformation are induced by external signals and forces, including growth receptors, talins, kindlins and metal ions, as well as a result in changes to the cellular environment

Integrins are a family of 24 heterodimer surface receptors which are formed by the combination of 18α and 8β subunits [12]. Each subunit consists of a large extracellular domain, a single transmembrane helix, and a short unstructured cytoplasmic tail [13, 14]. Integrins can exist in three major conformations: bent (inactive), extended (active), and a high affinity hybrid conformation, reflecting their ability to switch from inactive to ligand-bound forms [15, 16]. Structurally, integrins resemble a “head on two legs”. Wherein the α subunit contains a seven-bladed β propeller, which is connected to the calf-1 and calf-2 domains, which form part of the legs, where the head rests [17]. The β subunit consists of a hybrid domain, a plexin-sempahorin-integrin domain (PSI), four epidermal growth factor repeats rich in cysteine, the Metal-Ion Dependent Adhesion Site (MIDAS), and an adjacent site to the MIDAS (AMIDAS) [17, 18]. Nine variations of the α-subunit (e.g., α1β1, α2β1, αMβ2) [13] have an extra inserted segment, called the α-inserted domain (α-I domain), which is inserted between the β-propeller, that aids with ligand binding via a Rossman fold and a specialized MIDAS motif [18–20]. In integrins without the α-I domain, the β-I subunit packs closely to the α-dependent β-propeller, with the primary ligand binding site (β-MIDAS) lying on its surface, where the AMIDAS was found to regulate ligand-binding [21]. Lastly, the α helix is found to be in an orthogonal position with respect to the plasma membrane while the β-subunit helix assumes a slanted position [22, 23]. Figure 1 illustrates the structural organization of integrins.

### Integrin activation

Activation of integrins relies on conformational changes that are regulated by both internal and external forces. In its resting state, the head of the subunit bends towards the membrane, keeping affinity low for ligand binding. The binding of cytoskeletal proteins such as talins and kindlins or signaling proteins such as α-actinin and vinculin to the β tail causes separation of the tails and extension of the ligands, increasing affinity [24, 25]. External forces such as fluid shear stress or internal mechanical tension control integrin activation through bidirectional signaling [26]. Integrins can also cluster to increase avidity, which is commonly seen in the formation of immune synapses [25]. Parallelly, integrin trafficking is crucial for regulating activation of integrins by controlling the spatial and temporal availability of integrins at the plasma membrane. This trafficking redirects integrins to specific membrane regions, optimizing interactions between the ligands and cytoskeletal/signaling proteins, which ultimately facilitates the transition from inactive to active state [27].

### Integrin function

Functionally, integrins are classified based on their ligand binding preferences. This includes RGD binding (e.g. αvβ3, αvβ1, αvβ6), leukocyte-specific binding (e.g. α9β1, α4β1, αMβ2), collagen-binding (e.g. α1β1, α11β1, α10β1), and laminin-binding integrins (e.g. α1β1, α2β1, α3β1) [1, 28–31]. RGD binding integrins primarily bind to ECM molecules such as fibronectin, vitronectin, osteopontin, which act as ligands for cellular receptors, and transforming growth factor β (TGFβ). They play an important role in the remodeling of the extracellular matrix (ECM), wound healing, the activation of TGFβ, angiogenesis, modulation of inflammation, immune activation, cell migration, and platelet function [29].

Leukocyte-specific integrins are primarily involved in regulating inflammation, and recruit leukocytes such as neutrophils, basophils, eosinophils to the site of the infection. Here, adhesion glycoproteins, such as selectins, are expressed on leukocytes and can bind with ligands on vascular endothelial cells to initiate fast rolling. This allows integrins to bind with specific ligands such as ICAM-1, ICAM-2, and vascular cell-adhesion molecule (VCAM-1).Through this process, integrins are able to modulate the rolling, adhesion, and transmigration of leukocytes. Additionally, leukocyte-specific integrins aid in phagocytosis, modulation of inflammation, reactive oxygen species (ROS) production, cytokine production, and initiation of apoptosis [32].

Collagen-binding integrins recognize Glycine-Phenylalanine-Hydroxyproline-Glycine-Glutamic Acid-Arginine (GFOGER)-like sequences while also binding to fibrillar collagens (type I-XVI) [28]. They are commonly expressed in T-cells, fibroblasts, chondrocytes, keratinocytes, myeloid cells, endothelial cells, epithelial cells, and mesenchymal stem cells. These integrins play a key role in maintaining structural integrity, preventing fibrosis, promoting tissue and skeletal development [33, 34]. They also play a role in platelet metabolism, where they stabilize the thrombi while also minimizing bone loss, in addition to promoting inflammation in T-cells [32, 35].

Laminin-binding integrins are transmembrane glycoproteins that mediate cell adhesion to laminin-rich basement membranes and regulate key processes including cell migration, proliferation, survival, and tissue organization [36]. Some laminin integrins functionally overlap with collagen- or RGD-binding integrins. These integrins are widely expressed in epithelial, neural, vascular, and muscle tissues, where they maintain basement membrane integrity and support tissue-specific functions [32, 36, 37]. Dysregulation of laminin-binding integrins has been associated with impaired tissue organization and developmental defects, underscoring their importance in maintaining structural and signaling homeostasis [32, 37].

The conformation of an integrin is largely responsible for its function, and the orientation of each composite unit relative to the others defines the nature of the conformation. The three possible conformations are termed as bent, hybrid and open, which are visualized in Fig. 1 (L-R, respectively). Changes in conformation are induced by external signals and forces, including growth receptors, talins, kindlins and metal ions, as well as a result in changes to the cellular environment.

Integrins occupy a key position at the interface between cells and the ECM, where their regulated activation allows cells to sense and respond to biochemical and mechanical cues. Through this signaling role, integrins coordinate adhesion-dependent processes that mediate cell behavior and tissue organization. When integrin function is disrupted, these same mechanisms are altered to promote CSC hallmarks such as plasticity, stemness and therapy resistance while sustaining interactions with the TME. These cancer-associated functions of integrins are examined in the following section.

## Integrins in cancer

**Fig. 2:**
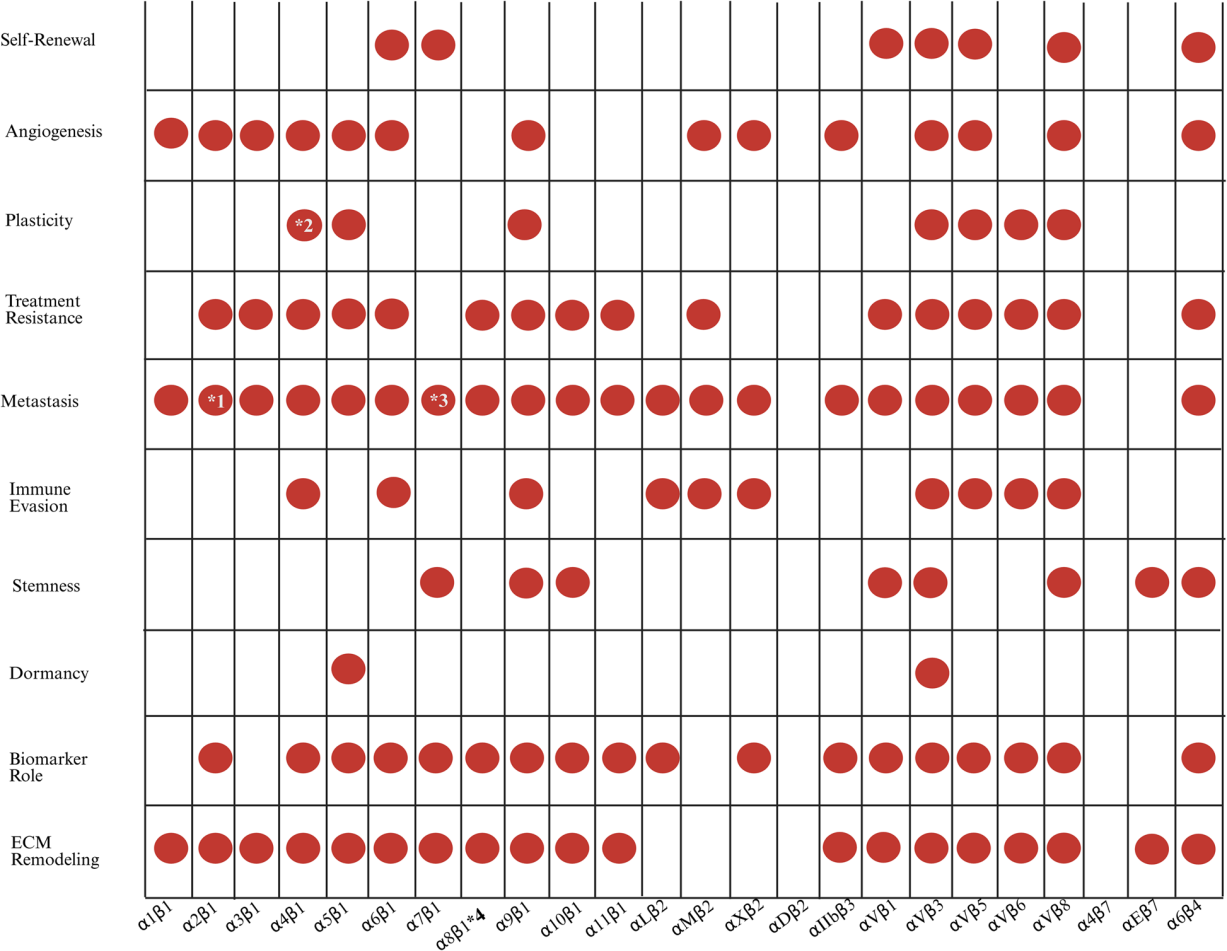
Matrix representation of the functional roles of integrins heterodimers in cancer. (Created with Biorender.com) In Figure 2, each column represents a specific integrin, labeled along the x-axis. Each row indicates a key-cancer related process, including self-renewal, angiogenesis, plasticity, treatment resistance metastasis, immune evasion, stemness, dormancy, biomarker utility, and ECM remodeling. Red dots indicate reported involvement of a particular integrin in the respective process. *1-The integrin α2β1 can play a dual role in cancer metastasis, either promoting or inhibiting it [38]. *2- For α4β1, the plasticity association was supported by evidence showing that nanoscale presentation regulates tumor plasticity [39]. *3- The integrin α7β1 has been found to play a suppressive role in melanoma metastasis when observed in murine models [40]. *4- The integrin α8β1 has been found to have a context dependent in cancer depending on the tumor type [41]

Integrins are important biomarkers in cancer, and their expression patterns differ across cancer subtypes. For example, the cancers with the highest concentration of integrins are head and neck cancer, stomach cancer, esophageal cancer, glioblastoma (GBM), and bile duct cancer**.** Cancers which have a lower concentration of integrins with respect to healthy normal tissue include lung cancer and kidney cancer [3]. Integrins are involved in promoting tumor stemness, the self-renewal and plasticity of CSCs, as well as enhancing drug resistance in several tumors, thereby making them essential hallmarks of various cancers. Figure 2 illustrates the 24 integrin heterodimers and their associated roles in tumors. The role of integrins in the aforementioned functions is discussed in the subsequent sections.

### Role in cancer stemness

Cancer stemness refers to the phenotype of stem cells for certain types of cancer cells, and it plays a critical role in multiple aspects of tumorigenesis [42]. CSCs are a group of tumor cells which exhibit bidirectional signaling, a property that allows them to respond to both healthy and damaged tissue microenvironments [8]. As a consequence, CSCs are highly adaptable and exhibit improved survival. Thus, they contribute to key tumor hallmarks, including tumor differentiation, initiation, self-renewal, metastasis, treatment resistance and tumor recurrence [43]. Integrins, through their role in modulating cellular growth, differentiation and inflammation, are able to function as genotypic markers for CSCs, in addition to being key molecular regulators for these processes [44].

Historically, certain cell surface markers have been used to identify certain CSCs, based on the assumption that CSCs retain phenotypic features of stem cell populations. One widely used example is the use of cluster of differentiation 34-positive/38-negative (CD34^+^CD38^−^) as markers for acute myeloid leukemias (AMLs) in clinical studies and cell cultures [42]. However, these markers have been found to regularly tag normal SCs, lacking specificity due to expression across a wide range of tissue types. Integrins have emerged as a favored marker for CSCs due to their membrane location and active role in tumorigenesis [45]. Among these, the laminin-binding integrin α6, which is commonly expressed in both neural and embryonic SCs. In GBM, cells positively expressing α6^+^ allow for multi-lineage differentiation and self-renewal, thereby allowing for the establishment of α6^+^ as a CSC marker. A combination of the cell surface receptor CD133 and integrin α6 was found to identify GBM stem cells (GSCs) in a much more efficient manner [46].The integrin α6 is also found to be upregulated in other cancers such as colon cancer, breast cancer (BC), and prostate cancer [42].

Other integrins have also been linked to CSC phenotypes. For example, Ming et al. observed that integrin α7^+^ was found to be a marker for esophageal cancer, where expression of α7^+^ showed increased signs of self-renewal, chemotherapy resistance, differentiation, and stemness. Upregulation of α7^+^ in esophageal tissues is correlated with lymph node metastasis, poor differentiation, and poor prognosis, likely mediated via the focal adhesion kinase (FAK) signaling pathway [47]. Recent studies have reported that the integrin β4 is a prominent marker in lung cancer, where it promotes therapy resistance, propagation of the tumor, and self-renewal [48]. Similarly, integrin β3 has been shown to activate stemness in human-epidermal growth factor receptor 2 (HER-2)-positive BC cell lines by activation of the Notch signaling pathway. Activation of this pathway drives CSC traits, including invasive growth, the ability to self-renew, and resistance to standard treatments. When integrins are overexpressed, stemness-related markers sex-determining region Y-box 2 (SOX2), NANOG, and others, become upregulated, this leads to the further resistance to trastuzumab. Blocking integrin β3 activity through the use of antagonists like Cilengitide has been shown to lower CSC marker expression, suggesting a possible route that may help overcome therapeutic resistance [49]. Additionally, integrin α4 also appears to hold prognostic and immunotherapeutic value, as it has been found to potentially act as a biomarker in cancers such as gastric cancer. Some studies suggest that it may contribute to CSC-like behavior through pathways such as the Hedgehog and phosphoinositide 3-kinase/protein kinase B/mammalian target of rapamycin (PI3K/Akt/mTOR) pathway, although its exact role is unknown [50].

Collectively, these findings indicate that integrins do not merely serve as surface markers for CSC identification but function as active regulators of stemness-associated signaling. By linking niche adhesion to pathways such as FAK, Notch, and PI3K/Akt, integrins translate extracellular cues into transcriptional programs that sustain self-renewal, plasticity, and therapy resistance. This dual role as both identifiers and functional drivers positions integrins as central mediators of CSC maintenance rather than passive phenotypic labels.

### Role of integrins in CSC Function

#### Integrins as promoters of niche anchoring and self-renewal

Integrins play an important in maintaining CSC function by mediating anchorage to the tumor niche and regulating self-renewal signaling pathways. This interaction aids in the transmission of biochemical and mechanical signals, which promote key properties of CSCs [8].

A study by Hamerlik et al. showed that growth factor signaling and integrin-mediated adhesion work together to maintain CSC populations within tumor niches. The study concluded that in GBM, autocrine vascular endothelial growth factor (VEGF)-VEGFR2-Neuropilin-1 (NRP-1) signaling was linked with the promotion of GSC viability and tumor growth. NRP-1 interacts with the integrin α6, the latter of which is a key moderator in the moderation of adhesion to perivascular niches rich in laminin. The integrin-NRP-1 complex promoted anchorage to the vascular basement membrane, a key aspect of niche retention that supports GSC survival, tumor growth and therapy resistance [51]. Parallelly, integrin α7 has also gained attention as a functional marker of GSCs. It mediates their adhesion to laminin, which, in turn, encourages tumor proliferation, invasion, and stemness [52].

In squamous cell carcinomas (SCCs), tumor-initiating stem cells are maintained through overlapping integrin-mediated mechanisms. A 2011 study showed that SCC tumor-initiating stem cells demonstrated higher levels of the integrin α6 and β1 subunits. The β1 subunit was found to form a signaling complex with FAK, which is key to the activation of pathways related to self-renewal and survival. The β1-FAK axis is controlled through TGFβ signaling, which aids in CSC maintenance. Disruption of the FAK or TGFβ pathways has been shown to disrupt the tumor-initiating capacity of SCC stem cells, suggesting that these pathways depend on each other for proper function [53, 54]. Together, integrins α6 and β1 appear to regulate the proliferation of tumors, their stemness, and epithelial plasticity [54].

In triple negative BC (TNBC), CSCs use integrin α6β1 to interact with laminin-rich niches. This interaction allows for cooperative signaling with the VEGFR-NRP-2 complex [55, 56]. As a result, FAK-mediated rat sarcoma (Ras)/mitogen-activated protein kinase kinase (MEK)-extracellular signal-regulated kinase (ERK) signaling is activated, leading to transcription of the Hedgehog effector glioma-associated oncogene homolog 1 (GLI1). GLI1 then drives the expression of B-cell-specific Moloney murine leukemia virus integration site 1 (BMI-1), which strengthens expression α6β1 and NRP-2, and establishes an autocrine loop that maintains both retention to the niche and CSC self-renewal [56].

In parallel, integrin α6β1 binds particularly to laminin-511, a matrix component regulated by the Hippo pathway effector transcriptional coactivator with PDZ-binding motif (TAZ). This integrin-laminin interaction anchors breast CSC while maintaining their tumorigenic state [57].

Similar mechanisms are also observed in bladder cancer. Here, the α6β4 integrin forms a functional axis with laminin to activate Notch signaling, which contributes to CSC maintenance and tumor progression [58]. These examples highlight the role of integrins in bridging niche adhesion to stemness-associated signaling.

Taken together, these findings indicate that integrins function as key mediators linking laminin-rich niche adhesion to stemness-associated pathways such as FAK, Notch, and Hedgehog pathways. Through these interactions integrins help maintain self-renewal and tumor-initiating potential. Accordingly, niche retention and stemness regulation seem interconnected rather than being separate CSC processes.

#### Integrins as modulators of plasticity in cancer stem cells

Phenotypic plasticity refers to the ability of cells to transition between both differentiated and stem-like states reversibly [59]. This feature of CSCs plays a significant role in tumor progression and therapy resistance. Plasticity is normally classified in to three categories, dedifferentiation, trans differentiation, and blocked differentiation [3].

A central manifestation of cancer cell plasticity is epithelial-mesenchymal transition (EMT), along with its partial and reversible forms collectively referred to as the epithelial-mesenchymal plasticity (EMP), which are fundamental to cancer cell aggression and invasive behavior [60]. Integrins are key regulators of EMT by coupling ECM engagement to intracellular signaling pathways that destabilize epithelial junctions and promote mesenchymal traits [8]. For example, activation of β1- and αv-containing integrins enhances FAK and Src signaling, leading to disruption of E-cadherin–β-catenin complexes and therefore promoting migratory and invasive behavior in tumors [61].

Tumor cells can be converted to CSCs, via a process termed de-differentiation. De-differentiation is reversible, making it a direct result of the plasticity of CSCs. For example, in colon cancer, integrin β1 enables the differentiation of non-stem tumor cells into CSCs. This is mediated through a signaling cascade involving the F-actin/tripartite motif-containing protein 11 (TRIM11)/phosphofructokinase (PFK)/hypoxia -inducible factor 1-α (HIF1α) axis. Disrupting integrin β1 impaired the dedifferentiation pathway [62], highlighting the crucial role of integrins in ECM-driven plasticity.

Integrins have also been found to control plasticity by interacting with various growth factors. In cutaneous SCCs, Lopez-Cerada and colleagues showed insulin-like growth factor-1 receptor (IGF1R) signaling induced epithelial-mesenchymal plasticity (EMP) through activation of the integrin αv. This axis allows epithelial cells to respond to signals coming from the TME, especially TGFβ, which encourages them to adopt a phenotypic shift towards mesenchymal states. Interestingly, blocking of IGF1R was found to reduce integrin αv expression, which prevents the generation of mesenchymal cancer cells. However, not all stem cell populations are affected, with existing mesenchymal stem cells usually left unaffected. This highlights that the IGF1R-αv axis is extremely important for the initiation of the EMP, while also being a potential target for therapies [63].

Beyond observable changes in the state of the cell, CSC plasticity also includes metabolic plasticity, which is known to be crucial for the survival of tumor cells under harsh conditions such as nutrient deprivation, or hypoxia. In non-small cell lung cancer (NSCLC), nutrient stress was found to upregulate αvβ3 signaling through the β3 subunit. While nutrient deprivation typically activates the stress sensor AMP-activated protein kinase (AMPK), stress induced activation of αvβ3 signaling via sarcoma (Src) kinase was found to stimulate sustained AMPK expression. [64, 65]. This sustained expression alongside mediation by peroxisome proliferator-activated receptor-gamma coactivator 1α (PGC1α) aids in converting the primary metabolic dependency from glycolysis to oxidative phosphorylation [64], highlighting the role of integrins in tumor cells to adapt their metabolism under stress.

These findings show us that integrins are important regulators of CSC plasticity by translating ECM and growth factor signals into pathways that govern EMT, dedifferentiation, and metabolic adaptation. Rather than simply supporting adhesion, integrins enable dynamic state transitions that allow tumor cells to shift between proliferative, invasive, and stress-adapted phenotypes.

#### Exosomal integrins and their role in cancer progression

Exosomes are extracellular vesicles (EVs) with a diameter of 30–130 nm that are secreted from cells and provide extracellular communication. Several studies have shown that integrins found in tumor-associated exosomes can aid in cancer progression [66].

In hepatocellular carcinoma (HCC), myofibroblast-derived EVs have been shown to promote CSC function by transferring integrin α5 to tumor cells. Upon uptake of α5-containing EVs in tumor cells, integrin α5 activated the Src family kinase (SFK), Yamaguchi sarcoma viral oncogene homolog 1 (YES1), through conformational changes and autophosphorylation. Activation of YES1-driven pathways such as yes-associated protein 1 (YAP1) and ERK1/2 was found to enhance stemness in tumor cells [67–69]. Disruption of integrin α5 expression or YES1 activation attenuated CSC features, such as increased tumor initiation in vivo and elevated treatment resistance. Integrin α5 was also found to require heterodimerization to integrin β5 to exert its function. Integrin β5 interference was found to impair α5-derived stemness, further research, however is required to understand the full molecular mechanism. This study highlights how stromal-derived integrins, when delivered through EVs, can directly modulate CSC behavior [69].

A similar mode of communication is also observed in the immune compartment in the TME. A recent study conducted in 2023 discovered that, in NSCLC, M2-like macrophage -derived exosomes promote metastatic potential through delivery of integrin αvβ3 to tumor cells. Delivery of integrin αvβ3 to the tumor cells was found to activate FAK signaling, enhancing NSCLC cell invasion and migration. Although the study did not directly assess the resident CSC population, acquisition of migratory behavior, invasiveness, and treatment resistance are known CSC characteristics. Additionally, blocking the integrin weakened cell migration and invasion, highlighting the role of exosomal integrin transfer in promoting tumor cell metastasis [70].

The clinical relevance of exosomal integrins has also been demonstrated in clinical cohorts of patients with lung cancer and brain metastases. In this setting, circulating exosomal integrin β3 was identified as a prognostic biomarker associated with poor survival and increased intercranial brain failure, following whole-brain radiotherapy. Elevated integrin β3 levels in plasma-derived EVs also correlated with brain-specific relapse, independent of characteristics such as age, histology, and number of metastases. These findings are highly suggestive that integrin β3 mediates brain-tropic metastasis, which has been linked with CSC function [71, 72].

In conclusion, these observations suggest that integrin signaling in CSC regulation includes EV-mediated communication. By transporting integrins between stromal, immune, and tumor cells, EVs allow integrins to operate throughout the TME. This distribution of integrins may influence tumor cell behavior and adaptation to metastatic potential.

#### Integrin trafficking and its role in promoting CSC behavior

Integrin trafficking, which includes internalization, recycling, and degradation of integrin receptors, helps regulate drug resistance, stemness and metabolic reprogramming-functions that are commonly associated with sustaining CSC behavior [73, 74].

Derby et al. found that tumor cell invasion is driven by enabling macropinocytic internalization of integrins at the growth-cone-like structures located at the tips of tumor microtubes (TMs). TMs are long, thin neurite-like projections that are enriched in GSCs. They enable dynamic interaction with the surrounding brain parenchyma, while also contributing to treatment resistance and tumor cell infiltration into key neural structures [75, 76]. Ataxia telangiectasia and Rad3-related protein (ATR)-dependent macropinocytosis supports the turnover and trafficking of integrins that is required for active migration of tumor cells. Additionally, inhibition of ATR disrupted GBM invasion both in vitro and in vivo, while overexpression of ATR improved tumor cell motility. These findings reinforce the role of integrin trafficking, especially through macropinocytosis, which plays a role in defining CSC characteristics such the regulation of cancer invasion and resistance to treatments [76].

A study by V.T. Duong et al. uncovered that a signaling axis involving Ras-related protein Rab-11 (RAB11), Munc13-4(UNC13D), and FAK plays an important role in pancreatic cancer, where it modulates endosomal recycling and focal adhesion (FA) which in turn drives invasive behavior. The protein UNC13D, facilitates the recruitment of FAK and also the phosphorylation of RAB11-positive recycling endosomes. These interactions promote the breakdown of FA, which encourages cell migration. Importantly, integrins and growth factor receptors like cellular-mesenchymal epithelial transition factor (c-Met), are both taken into the cell and delivered to endosomes, where they sustain the signaling of FAK. Inside these endosomes, FAK is bound to integrins through its four-point-one, ezrin, radixin, moesin (FERM) domain. This process, supports the survival of cells and their migration, both of which are key characteristics of CSCs [77, 78].

These findings demonstrate, the studies indicate that integrin trafficking influences the ability and localization of integrins within tumor cells. Through effects on the internalization and recycling of integrins, trafficking processes may affect how cells adjust adhesion and signaling during migration and stress conditions. These observations indicate a possible contribution of trafficking dynamics to CSC behaviors related to survival and motility.

#### Immune evasion and drug resistance

In recent years, integrin signaling has been found to be strongly associated with immune evasion and therapy resistance across multiple tumors, features that are frequently linked with CSC function [3, 8].

In a study using melanoma and BC models, regulatory T-cells (T_reg_) were shown to enable immune evasion through expression of integrin αvβ8 which aids in activation of TGFβ secreted by tumor cells in the TME. TGFβ is initially secreted in an immunologically inert form, and its activation occurs through the removal of the latency-associated peptide (LAP), which shields the contact/receptor sites [79]. Integrin αvβ8 binds to LAP via its RGD-recognition motif, facilitating the activation of TGFβ, making it capable of interacting with TGFβR1/2. This interaction leads to SMAD2/3 phosphorylation, leading to the suppression of cytotoxic activity, and therefore aiding tumor survival. The absence of the β8 integrin chain in T_regs_ impairs TGFβ signaling, leading to the control of tumor growth through cytotoxic function. This highlights the key role of integrin αvβ8 as a critical regulator in immune evasion [80].

Similarly, GSCs have been shown to evade surveillance by natural killer (NK) cells by activating TGFβ signaling through integrin αv-mediated mechanisms. A study by Gruber et al. established that direct contact between NK cells and GSCs activate αv integrin-mediated secretion of inert TGFβ. Matrix metalloproteinase 2 (MMP2) and MMP9, predominantly secreted by GSCs, cleave TGFβ into its active form. Active TGFβ signaling can suppress NK cytotoxicity via SMAD2/3 phosphorylation, leading to phenotypic expression and loss of effector function. Findings also showed that αv integrin expression on GSCs is required to initiate the axis. Clustered regularly interspaced short palindromic repeats (CRISPR)-mediated knockdown of αv integrin or inhibition using cilengitide disrupts TGFβ production, preserving NK function. This study highlights the αv integrin-TGFβ axis as a critical mechanism for CSC-mediated immune evasion [80].

The integrin αvβ3 was found to promote radio resistance. A study on cervical cancer patients revealed that individuals with enhanced αvβ3 content had higher rates of metastasis, along with a worse prognosis [81]. Radio resistance through αvβ3 was found to be caused by regulation in survivin levels. High expression of survivin in CSCs is linked to radio and chemo resistance, with studies showing that GBM cell lines have higher levels of survivins [82, 83]. Lower expression of survivin was found to be associated with higher radiosensitivity. The integrin αvβ3 maintained survivin expression after exposure to irradiation, which would have been inhibited if αvβ3 was absent [84, 85].

In oral squamous cell carcinoma (OSCC), both drug resistance and tumor recurrence are linked closely with CSC populations. For example, a study conducted in 2022 identified keratin 17 (KRT17) as a regulator of OSCC stemness and chemoresistance, through modulation of integrin-dependent signaling. KRT17 was found to bind to plectin, a cytoskeletal protein, which results in the activation of integrin β4/α6, increased phosphorylation of Src, ERK, and FAK, as well as stabilization and nuclear translocation of β-catenin. Activation of this signaling cascade resulted in enhanced OSCC stemness, along with elevated expression of epidermal growth factor receptor (EGFR) and CD44, which have been associated with promotion of CSC phenotypes in TNBC [86, 87].These effects were reversed when KRT17 was targeted with miR-485-5p, a tumor-suppressive micro-RNA (miRNA). Additionally, the study demonstrated that dasatinib, a Src inhibitor, sensitized OSCC cells to platinum-based chemotherapies through disruption of the signaling cascade [86].

In ovarian cancer (OC), platinum resistance and tumor recurrence have become increasingly attributed to ovarian cancer stem cells (OCSCs) that persist following treatment. In the TME, the ECM enriched with fibronectin promotes encourages the clustering of integrin β on the cell surface. This clustering activates the sustained release of integrin-linked kinase (ILK) which physically activates the Wnt receptor frizzled-7 (Fzd7). The activation of Fzd7 improves β-catenin signaling and adenylate kinase (AK) phosphorylation, both of which are important for the maintenance of CSC and the survival, maintenance and spheroid formation of tumors [88, 89]. Particularly, ILK was enriched in aldehyde dehydrogenase (ALDH) +/CD133 + OCSCs, with its inhibition through cpd-22 which dysregulate the clustering of Fzd7, impairing Wnt responsiveness, and CSC sensitization to carboplatin-induced apoptotic cascade [89].

These findings indicate that integrins contribute to CSC survival through both intracellular pathways and by reshaping the immune and therapeutic responses. Through regulation of survival signaling and adhesion-dependent resistance mechanisms, integrins are able to protect tumor cells from cytotoxicity. This highlights integrins as mediators of immune evasion and therapy resistance.

While integrin signaling is frequently framed as a regulator of intrinsic CSC properties such as self-renewal, plasticity, and therapy resistance, this perspective alone is insufficient. CSC phenotypes emerge from continuous, bidirectional interactions with the ECM and stromal components of the TME. Additionally, integrins not only interpret ECM-derived signals but also actively reshape the architecture of the matrix and stromal behavior, thereby reinforcing malignancy beyond CSC populations. Understanding integrin function in cancer therefore requires expanding analysis from CSC-centered processes to integrin-driven mechanisms in the TME and ECM.

### Role in extracellular matrix and tumor microenvironment

#### ECM remodeling and invasion

The progression of cancer from being localized to invasive occurs through a series of changes in cellular adhesion. An important event in this transition is the loss of E-cadherin-mediated intercellular junctions, which is accompanied by an increase in integrin-dependent adhesion to the ECM [90, 91]. This shift allows cancer cells to detach from the basement membrane and the neighboring epithelium cells, which is an important step towards the promotion of invasion and metastasis in tumors [91].

Integrins which contain the β1 heterodimer were found to activate FAK and SFKs, which phosphorylate the E-cadherin-β-catenin complex. This process destabilizes the cell junctions and thereby promotes the invasion and migration of the tumor cells [92]. The dual nature of signaling in integrins also disrupts cell–cell adhesion which enhances the contractability of actomyosin, while also destabilizing the E-cadherin junction mediated by the Fak and Src signaling pathways [93]. Src signaling was also found to induce E-cadherin endocytosis, which weakens adherens junction while also promoting the invasion and migration of tumor cells [4]. Importantly, adhesion to fibronectin through integrins provides negative feedback that discourages E-cadherin expression, and thereby promotes the detachment of cells and their resulting motility [94]. Additionally, restoration of β1 expression in cells that did not previously express it has been found to decrease the extent of contact between neighboring cells, which compounds the effect of cell dispersion discussed earlier. This observation illustrates the role integrins play in encouraging invasive behavior [91]. Once tumor cell detaches, they rely on the interactions between the integrin and ECM to coordinate adhesion and invasion. Integrins also recruit MMPs to remodel the ECM and aid in tumor mobility [95, 96]. For example, in glioma, integrin α5β1 interacts with MMP2, which activates interleukin-6 (IL6)/signal transducer and activator of transcription 3 (STAT3) signaling pathways. These results suggest that this interaction supports both the survival and invasive potential of cancer cells [96]. Similarly, in colon cancer, MMP2 can cleave the β1 integrin, disrupting the adhesion between the cell and the ECM while also preserving downstream signaling. This cleavage improves the trafficking of integrins and encourages cell motility which allows tumor cells to remodel adhesion sites and invade through the ECM [97].

These findings indicate that integrins facilitate invasion by strengthening tumor-ECM adhesion and by coordinating junction disassembly and proteolytic remodeling. By linking cytoskeletal signaling to MMP activity and ECM engagement, integrins remodel adhesion to support direct migration. Therefore, we can assume that integrins are regulators of mechanical attachment and matrix restructuring.

#### Mechanotransduction in ECM-related tumorigenesis

Due to dual nature of signaling in integrins, they also function as mechanosensors of ECM stiffness, and are responsible for the reinforcement of downstream signaling that promotes tumorigenesis. Additionally, the activation of integrins through matrix-derived signaling have been shown to support both pro-tumor signaling and their progression [8]. Additionally, a study carried out in 2023 revealed that matrix rigidity drives the collective tumor invasion in BC through a mechanotransduction loop that involves both integrins and periostin (POSTN). In stiff ECM conditions, YAP signaling activates cancer associated fibroblasts (CAFs), which increases the secretion of POSTN, this leads to greater collagen crosslinking and an increase in ECM stiffness [98–100]. This remodeling specifically triggers integrin signaling, especially through αvβ3 and αvβ5 integrins, in neighboring tumor cells which encourages the activation of the FAK-ERK cascade, this leads to remodeling of the cytoskeleton through cell division control protein 42 (Cdc42) and Ras-related C3 botulinum toxin substrate 1 (Rac1)-mediated mechanisms. These pathways even control actin polymerization, leader cell formation, and traction force generation, all essential steps for coordinated tumor invasion [100, 101]. In clinical practice, tests showed that high levels of POSTN and collagen was linked with an increase in tumor recurrence in BC, thus supporting the possible importance of the YAP-POSTN-integrin axis in tumor invasion and its progression [100].

Recent work has also highlighted matrix stiffness as an important contributor in promoting perineural invasion, which is the spread of tumor cells along the nerves of the body [102]. In BC, stiffened ECM conditions promote invasiveness and exhibit higher expression of neutrophic factors, such as nerve growth factor (NGF), which attract and interact with other nerve fibers. These nerve fibers become involved with several stromal cells in the tumor microenvironment (TME), thereby affecting tumor characteristics such as growth, invasion, metastasis, and drug resistance [103, 104]. Integrin β1 acts as a central mechanosensor, primarily acting via translating rigidity changes of the ECM into intracellular signals through activation of the FAK-YAP pathway. The aforementioned pathway promotes the activation of epithelial mesenchymal transition (EMT), while simultaneously encouraging the upregulation of pro-invasive genes and the secretion of NGF, which leads to the creation of a neutrophic TME that is important in nerve infiltration. Disrupting the β1-FAK-YAP axis showed a reduction in both the invasive and neutrophic phenotypes that are normally induced by a stiff matrix. Underscoring the role of integrin β1-mediated stiffness as a possible regulator in the perineural invasion of BC [104].

These studies indicate that integrins translate ECM stiffness into pro-tumorigenic signaling. Interactions with YAP and FAK as well as cytoskeletal regulators, integrins are able to convert mechanical cues into signaling pathways that encourage survival and metastatic potential. This supports the view that tumors are influenced by the interaction of integrins with both biochemical signaling and mechanical properties of the TME.

#### Glycosylation-led modulation of ECM-integrin functions

Post-translational modifications, such as glycosylation, critically modulate integrin-ECM interactions, altering cancer cell activity [105, 106]. Glycosylation is a process in which sugar molecules attach to proteins, thereby altering integrin signaling and function. This process negatively affects their ability to bind to the ECM and drive signaling programs such as tumor cell growth and survival [107]. Protein glycosylation commonly includes N-linked glycosylation and O-linked glycosylation. N-linked glycosylation is a process where a carbohydrate, such as a glycan, is attached to the nitrogen atom of an aspargine amino acid in a protein. O-linked glycosylation involves a carbohydrate attaching to the oxygen atom of serine or threonine amino acid in a protein [108].

A study by Gardi et al., found that in lung cancer, post translational glycosylation of the ECM can be a key modulator of CSC behavior through β1 integrin-mediated mechanotransduction. Glucose-functionalized collagen (Glc-collagen) was found to increase the number of several cell populations, including CD133 + cells, lung CSCs, and its metastatic subset CD133^+^ C-X-C chemokine receptor type 4 (CXCR4) ^+^ epithelial cell adhesion molecule (EpCAM)^−^ cells. This happens via mimicking ECM glycosignatures, which enables the survival of slow-proliferating tumor cells [109, 110]. Enrichment of these cells was linked with greater tumorigenicity and metastatic potential in vivo. Glc-collagen was also found to promote the selective adhesion and activation of the β1 integrin, which, when activated, was upregulated in CSC subsets and formed heterodimers with integrin α1, which favored collagen binding. Inhibition of integrin β1 disrupted expansion of CD133 + and CD133^+^CXCR4^+^EpCAM^−^ CSC subsets on glycosylated matrices, leading to lesser display of CSC-like functions. This highlights that integrin β1-dependent signaling of ECM glycosylation dictates stemness and metastatic behavior [110].

In 2021, it was reported that integrin α2 was found to be an O- and N-glycosylated protein that facilitates ECM adhesion and promotes tumor cell adhesion and survival in collagen-rich TME of ovarian cancer. Glycoproteomic analysis has revealed 7 distinct N-glycosylation sites on integrin α2, including residues that regulate collagen and laminin binding. Site-specific mutagenesis of these glycosites can lead to disrupted integrin heterodimerization and inhibited focal adhesion. This leads to proteosome-mediated degradation of the integrin, and in turn increases apoptosis, bringing attention to the importance of glycosylation in integrin stability and tumor survival. In addition to N-glycans, O-glycosylation of integrin α2 has been associated with more aggressive cancer behaviors. This suggests that glycosylation plays a critical role in how integrins sense the ECM and mechanotransduction. Alterations in glycosylation patterns have been associated with tumor progression [111].

A study by S.Jain and colleagues investigated the diagnostic potential of integrin α3 glycosylation in epithelial ovarian cancer (EOC). Their analysis of patient-derived cysts and ascitic fluid showed cells expressing integrin α3 with sialylated Tn (STn) antigens demonstrated greater diagnostic accuracy in distinguishing benign and malignant cells than integrin α3 alone. Atypical glycosylation patterns, such as STn expression, emerge early in tumorigenesis, destabilizing integrins, and promoting immune evasion. Tissue specific expression of glycovariants such as STn on EOC, suggests integrin glycosylation can be used as liquid biopsy markers with clinical relevance [112].

Collectively, these observations suggest that glycosylation influences integrin-ECM interactions and downstream signaling behavior. By altering the stability of receptors, ligand binding, and pathway activation, glycosylation may affect metastatic capacity, survival and stemness. These changes also suggest that integrin glycovariants may have diagnostic and functional relevance.

#### Stromal crosstalk

Tumor-associated fibroblasts (TAFs)/CAFs and tumor-associated macrophages (TAMs) have been found to largely contribute to tumor progression [113]. It is known that TAFs play a role in the promotion of metastasis and invasion in several types of cancers. This is largely due to their myofibroblast-like traits, which generates a stiff ECM, eventually providing adhesion and mechanical forces for tumor growth [114, 115]. For example, ligation of integrin α2β1 to collagen has been shown to regulate lysyl oxidase expression in cardiac fibroblasts, presenting the possibility that a similar mechanism may operate in TAFs to promote collagen crosslinking and ECM stiffening [116, 117]. In addition to collagen remodeling, TAFs influence modulation of fibronectin arrangement through integrin αvβ3-dependent mechanisms. This fibronectin matrix arrangement can stiffen the ECM, and CAFs can even physically pull cancer cells directly from the primary tumor site, guiding invasion [118]. Importantly, responses of tumor cells to TAF-derived signals occur through integrin-dependent mechanisms. For instance, it was found that the CXCL12/CXCR4 signaling in TAFs promotes β1 clustering on tumor cells, activating the promotion of invasion through FAK activation [119]. Certain integrins, such as α2β1, expressed on tumor cells further initiate pro-invasive proteolytic cascades through stromal cell interactions [117]. For example, the interaction of integrin α6β1, expressed on pancreatic cancer cells, with urokinase plasminogen activator receptor (µPAR), which is expressed on stromal fibroblasts, has been shown to activate the µPAR-µPA-MMP2 proteolytic axis in stromal cells. This has been shown to enhance ECM degradation and enabling tumor invasion [120]. TAMs play a key role in helping tumors grow, especially by aiding cancer cell invasion and angiogenesis. Recent studies have found that integrins are important regulators of TAM-driven tumor growth. For example, when the ECM is rich in osteopontin, it can activate TAMs by binding to the α9β1 integrin. Once activated, these TAMs produce and release prostaglandin E2, a signaling molecule that encourages both endothelial cells, and cancer cells to migrate. This creates a microenvironment that fuels the growth of tumor and metastasis [117].

These findings suggest that integrins mediate the bidirectional crosstalk between tumor cells and the stroma population residing in the TME. Through regulation of ECM organization, chemokine-driven signaling, and proteolytic cascades, integrins promote tumor-stroma signaling that encourages tumor invasion and matrix remodeling. This demonstrates the role of integrins as a central factor in tumor-stroma interactions.

#### Dormancy, ECM and integrin crosstalk

Recent research has emphasized the importance of tumor dormancy in metastasis; wherein dormant cancer cells can later reactivate and contribute to the recurrence of these tumor cells. Studies have also indicated that the TME plays an important role in tumor dormancy regulation, particularly through the integrin signaling pathway [121].

For example, integrin-ECM interactions play a large part in controlling whether dormant tumor cells remain inactive or reactivate and re-enter the cell cycle. In a study by Bui et al. they found that genetic disruption of integrin β1 expression in mammary epithelial cells led to halted tumor progression and also induced tumor dormancy, this was done through mechanisms such as decreased proliferation, cellular senescence, and apoptosis induction. This integrin loss led to the activation of p53-dependent checkpoints blocking further growth [122]. Through p53 inactivation, reactivation of these dormant cells is possible, indicating that genetic changes in tumor cells, alongside changes in the stromal TME are crucial for cancer cell reactivation [122, 123]. Interestingly, cells deficient in the β1 integrin that were able to evade activation of dormancy showed higher levels of dense collagen deposition, infiltration of CAFs and a fibrotic ECM, possibly suggesting that integrin signaling alongside the TME is mandatory for tumor malignancy [122]. These dormant tumor cells also exhibit reduced immune surveillance, suggesting a temporary immunosuppressive niche that supports the survival of dormant cells [124]. This study highlights the role of β1 integrins as a regulator of dormancy, ECM rigidity, immune modulation, and CAF-driven fibrosis [122].

Research has also highlighted the importance of integrin-mediated ECM remodeling in the maintenance and reactivation of dormant tumor cells. A study by Barney et al., found that tumor cells surviving under serum-deprivation induced stress remodeled the ECM in BC by assembling a fibrillar fibronectin matrix, a process highly dependent on integrins αvβ3 and α5β1. These integrins mediate anchorage, while also cooperating with Rho-associated kinase (ROCK)-driven intracellular tension and autocrine TGFβ2 signaling to assemble a fibronectin matrix that promotes cellular quiescence, which is a key component of tumor dormancy, wherein tumor cells halt division to survive in a non-proliferative state [125, 126]. Among the two integrins, integrin α5β1 plays a key role in facilitation of focal adhesion and contractability, which are necessary for matrix organization. The process of cancer cells exiting from dormancy requires degradation of the matrix through MMP2. Moreover, blocking integrin signaling, ROCK, or TGFβ2 pathways have been shown to significantly disrupt the assembly of fibronectin, which impairs the survival of dormant cell population [125]. This study highlighted the key role of integrin-ECM signaling in dormant tumor cell survival.

In lung adenocarcinoma, chemotherapy-induced dormant tumor cells were found to secrete exosomes enriched in the integrin β6, which were taken up by surrounding fibroblasts. After uptake, the β6 integrin activated a krüppel-like factor 10 (KLF10)-dependent positive feedback loop, which engaged the TGFβ signaling pathway, driving the conversion of fibroblasts into CAFs. Activated CAFs become more contractile, and show increased levels of α- smooth muscle actin (α-SMA) expression. Greater ECM remodeling capacity, leads to stiffer tissue that supports the growth of tumors. Integrin β6 also triggers the TGFβ-KLF10 axis, which sustains the conversion of CAFs, while also aiding in the formation of a dormant tumor niche. This is done by enriching the ECM with collagen and fibronectin, both of which are known for their role in regulating tumor dormancy through integrin-mediated adhesion and mechanotransduction [4, 127, 128].

Together, these studies indicate that integrin–ECM signaling contributes to both regulation of tumor dormancy and reactivation. By influencing adhesion, organization of the matrix, and stromal signaling, integrins may help shape microenvironments that support non-proliferative tumor states or reactivation of tumors.

#### Influence of integrins in promoting tumor angiogenesis

Angiogenesis is an essential aspect of tumor growth and maintenance within the TME, as it sustains oxygen and nutrient availability within the tumor niche, due to which it has become a major target of therapies aimed at inhibiting blood vessel formation [129, 130]. Several integrins, such as the αv integrins and α5β1, have been found to be involved in the promotion of angiogenesis in tumors through regulation of the migration and proliferation of endothelial cells [131, 132]. However, it was observed that despite the deletion of the endothelial cell RGD-binding integrins (α5β1 and αvβ3/β5), there was no change in tumor angiogenesis, suggesting that there are other integrins that are complicit in the activation and promotion of tumor associated angiogenesis [131]. Pro-angiogenic effects have shown to be present in the integrins α2β1 and α1β1. Additionally, the integrin α4β1 and its ligand, VCAM-1, were found to play a large role in tumor vessel formation [133, 134]. Some integrins, such as αvβ3, were also found to upregulate angiogenesis using paracrine distribution, and this process occurs through the secretion of soluble factors that can lead to the stimulation of endothelial cells. This represents the importance of integrins in certain tumor cells, such as BC cells where the integrin α3β1 stimulates MMP9 [6] and cyclooxygenase 2 (COX2) expression [135]. In BC cells, the integrin α6β4 was also found to strengthen the expression of VEGFR, which supports both tumor survival and angiogenesis [136]. These findings highlight the role of integrin crosstalk in integrin-mediated angiogenesis in the TME.

These observations demonstrate that integrins participate in angiogenesis through endothelial cell mechanisms and tumor cell signaling. Through regulation of migration, adhesion and paracrine distribution, integrins influence vascularization within tumors.

This feature of CSCs plays a significant role in tumor progression and therapy resistance.

This section demonstrates that integrins operate at the core of cancer progression by integrating CSC-intrinsic signaling with extracellular matrix dynamics and tumor microenvironmental regulation. Rather than solely acting as markers or adhesion receptors, integrins coordinate stemness, plasticity, dormancy, immune evasion, and therapy resistance through continuous bidirectional signaling between tumor cells and their surrounding niche. These functions are highly context-dependent, and are shaped by composition of the ECM, stromal crosstalk, mechanical cues, and integrin trafficking, which together define integrin signaling output in tumors.

The functional centrality of integrins also explains the interest in them as therapeutic targets. Their convergence at CSC biology and microenvironmental remodeling makes them attractive points of intervention, however, the context-specific and adaptive signaling highlighted above likely contributes to the limited clinical success of integrin-based therapies to date. The following section explores current integrin-targeting strategies, as well as discussing the failures of previous attempts.

## Integrins in cancer treatment

In recent years, numerous studies have been exploring integrins as a possible target for treatments. Treatments targeting integrins have shown to be effective in inhibiting CSC-like properties such as drug resistance, immune evasion, plasticity, and self-renewal. Additionally, targeting integrins has also been found to disrupt TME dynamics, including ECM remodeling, angiogenesis, and stromal crosstalk. As integrins integrate signals from the ECM to regulate tumor behavior and niche function, their therapeutic inhibition holds great promise.

### Targeting Integrins in CSC function

#### Targeting niche anchoring and self-renewal

As discussed in section, 3.2.1., integrins contribute to niche anchoring by mediating the adhesion of CSCs to ECM components within the supportive microenvironments, where local biochemical and mechanical cues are transmitted intracellularly. Through these interactions, integrins influence signaling pathways which reinforce self-renewal, enabling CSCs to maintain an undifferentiated state while sustaining their tumor-initiating capacity [8]. Consequently, targeting integrin-mediated mechanisms between CSCs and their niches disrupt systems that sustain tumor-initiating potential and resistance to therapy [137].

An excellent example of how cancer cells shape their microenvironment comes from GBM. In this tumor, glioma-initiating cells (GICs) actively remodel their surroundings by secreting ECM proteins such as fibronectin and laminin [138]. In work done by Niibori-Nambu and colleagues, transcriptomic and proteomic analyses showed that during early differentiation triggered by serum, GICs increase the production of ECM components including collagen IV, fibronectin, and laminin α2. This remodeled ECM creates a niche that promotes further differentiation and GIC growth. An important component of this process is integrin αv, which is enhanced alongside fibronectin and supports adhesion-driven signaling which is critical for the proliferation and malignancy of GICs. Blocking this interaction with RGD peptides or antibodies which target αv interrupted the cells’ ability to anchor, while decreasing differentiation, slowing proliferation, and making the cells more chemo sensitive to the drug temozolomide. In mice, combining temozolomide with RGD treatment decreased the growth of tumors extending their survival. These findings highlight integrin αv as a potential g target in GBM treatment through its part in shaping the niche of [139].

A study from Crippa et al. employed an “early metastatic niche-on-a-chip” and explored the role of the β3 integrin in regulating BC extravasation. In the 3D model, inclusion of both neutrophils and platelets significantly increased transendothelial migration of MDA-MB-231 cells through enhancement of EMT marker expression and disruption of endothelial junctions [140–142]. Mechanistically, the β3 inhibition suppressed the Src-FAK-VE cadherin signaling cascade, limiting vascular endothelial cadherin (VE-cadherin) phosphorylation and nuclear translocation, which allowed for preservation of the endothelial barrier. Treatment with eptifibatide, a clinically approved β3 inhibitor, reduced platelet aggregation, tumor cell adhesion and invasion. Additionally, eptifibatide downregulated EMT associated genes such as talin and FAK in tumor cells, therefore limiting their invasive capabilities [142]. These findings position β3 integrins as a viable therapeutic targeting of early metastatic niche formation.

#### Targeting plasticity

Plasticity induced by hypoxia proves to be a barrier in effective cancer therapy, due to its facilitation of metastatic dissemination through remodelling of migratory mechanisms. A study from Boekhorst et al. demonstrated a critical mechanism, wherein hypoxia, through HIF stabilization, induces the phenotypic transition from collective or mesenchymal migration to blebbing amoeboid migration, in epithelial tumors such as BC [143]. Blebbing amoeboid migration can be defined as the migratory process in which cells such as immune cells or tumor cells utilize protrusions in the membrane to move through the environment [144]. Calpain-2, a calcium dependent cysteine protease that mediates the cleavage of talin-1, was found to drive plasticity in this environment. Talin-1 is a focal adhesion adaptor that is critical for activation of the β1 integrin. Widespread deactivation of integrins results in impairment of cell–ECM adhesion, thereby promoting the transition toward “eco-mode” amoeboid motility, which is maintained by a low-oxidative and low-glycolytic metabolic profile. Pharmacological inhibition of calpain activity, through an inhibitor such as PD150606, prevented deactivation of β1, highlighting the potential of the calpain-2-talin-1-β1 integrin pathway as a druggable regulator of tumor cell plasticity [143].

A recent study, used 3D cultures, to study the role of disintegrins, a group of proteins found in snake venom, in inhibiting tumor growth [145, 146]. DisBa-01, a high affinity integrin αvβ3 inhibitor derived from the venom of *Bothrops alternatus*, demonstrated potent anti-angiogenic and anti-migratory effects in TNBC and endothelial spheroids in both normal and hypoxic conditions. DisBa-01 impaired directional migration in both MDA-MB-231 TNBC cells and human umbilical endothelial vesicle (HUVEC)-derived endothelial spheroids, despite the latter exhibiting higher basal αvβ3 integrin expression. At the mechanistic level, DisBa-01 treatment downregulated VE-cadherin, an important mediator of endothelial plasticity [147], thereby suppressing sprouting in HUVECs [146]. These findings highlight how regulating TNBC migration and endothelial plasticity and positioning DisBa-01 makes for a promising therapeutic option for inhibition of tumor plasticity.

#### Targeting drug resistance and immune evasion

**Fig. 3:**
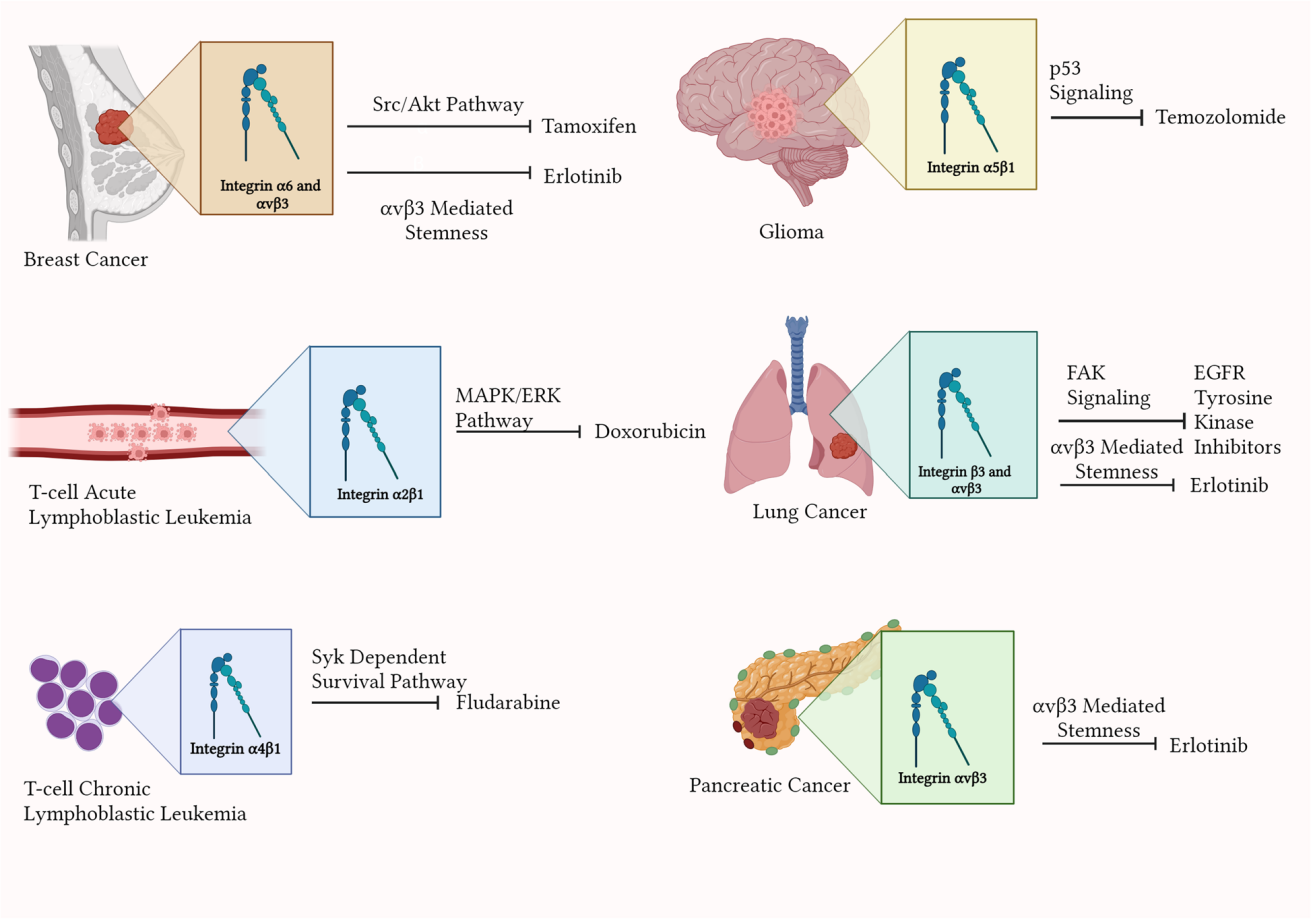
Integrins promoting treatment resistance in their respective cancers (Created with Biorender.com) Integrins possess a wide variety of roles in different cancers, with one integrin capable of performing different functions in different cancers. Figure 3 highlights the effects of various integrins on treatment resistance in several cancers. Integrins are capable of inducing treatment resistance through interfering with the cellular pathways targeted by the drug, or through inducing resistive cellular responses. Several important chemotherapeutic drugs, such as Temozolomide, are prone to integrin resistance and thereby, reduced treatment efficacy in gliomas

Key functions that serve as therapeutic targets include drug resistance and immune evasion, which, as discussed in previous sections, are both driven by integrin-mediated activity. Cheng and colleagues identified a signaling axis dependent on integrin β3, alongside identifying Kirsten rat sarcoma viral oncogene homolog (KRAS) as a regulator of pancreatic CSCs (FGβ3 cells) [148]. They also discovered that PAWI-2, a novel drug, can specifically target this pathway in FGβ3 cells, which are typically resistant to conventional therapies such as erlotinib. PAWI-2 disrupts the ability of these CSCs to survive and self-renew through the impairment of KRAS-driven downstream signaling [148, 149]. Mechanistically, PAWI-2 promotes the phosphorylation of the adaptor protein optineurin at Ser177, which facilitates its move into the nucleus, which in turn triggers G2/M cell-cycle arrest. This process simultaneously suppressed the activation of TANK-binding kinase 1 (TBK1), a kinase downstream of integrin β3-Ras-like protein B (RaIB)-KRAS, through a negative feedback mechanism, thereby inhibiting the pro-survival nuclear factor κβ (NF-κβ) pathway. By disruption of the β3-KRAS-NF-κβ axis, PAWI-2 effectively overcomes drug resistance and restores the efficacy of treatments such as erlotinib. When compared to bortezomib, PAWI-2 demonstrated greater synergistic effect with erlotinib, and provided targeted therapeutic strategy against drug-resistant pancreatic cancer [148]. Figure 3 illustrates therapeutic interventions affected by integrins across various cancers, highlighting the importance of targeting integrin-mediated drug resistance.

Recent studies have also demonstrated the key role played in integrins in helping tumors evade the immune system. One such study found that the integrin αvβ6 plays a key role in colorectal cancer immune evasion, which occurs primarily through the activation of the latent TGF-β and suppresses cytotoxic CD8^+^ T cell activity. This immune inactivation mechanism renders tumors resistant to immune checkpoint blockade therapy [150, 151]. Antibody-based inhibition of integrin αvβ6 sparked reactivation of T-cell response, significantly enhancing the efficacy of anti-programmed cell death protein 1 (PD-1) therapy in murine models, restoring treatment sensitivity in colorectal cancer. Additionally, αvβ6 inhibition achieved localized tumor immune activation, in contrast to systemic TGF-β blockades which can be associated with off-target toxicity [151].

The strategies described in 4.1 primarily target integrin-dependent signaling pathway that sustain CSC-associated traits such as: drug resistance, immune evasion, plasticity, and self-renewal. However, integrin activity in tumors also operates within the ECM and TME, where it influences the physical and biochemical conditions that support pro-tumorigenic phenotypes. Therefore, therapeutic approaches focused solely on CSC-intrinsic properties may prove to be insufficient to fully disrupt tumor progression. The following section discusses therapies targeting integrin functions involved in ECM remodelling and TME-associated processes. 

### Targeting Integrin-mediated ECM and TME

#### Targeting ECM Remodeling and invasion

Cancer cells exploit integrin signaling to alter the TME through MMPs and force-generating actomyosin networks that produce contractile forces to remodel the ECM**.** Targeting this integrin-mediated ECM remodeling axis offers an opportunity to restrain tumor invasion, stromal conditioning, and metastatic dissemination.

For example, a study conducted in 2020 detailed how β1 integrin signaling directly regulates ECM remodeling through control of trafficking and activation of membrane type-1-MMP (MT1-MMP), a membrane tethered protease that is critical for invasive cancer phenotypes. The study demonstrated that the β1 integrin engagement enhances the MT1-MMP phosphorylation at Thr567 via both EGFR and Src signaling, this promotes internalization and recycling to invadopodia, actin-rich protrusions which play a large role in ECM degradation. The β1-Src-EGFR axis plays a key role in the enhanced presence of MT1-MMP, while also increasing activation of MMP2 and MMP9, which implies further matrix degradation. Blocking of Src or EGFR signaling interrupted MT1-MMP phosphorylation and invadopodia formation, confirming that the β1 is a possible therapeutic target [152].

Recent studies have also identified integrin α11β1 as an important collagen receptor that is abundant in CAFs within desmoplastic tumors, including NSCLC, pancreatic ductal adenocarcinoma (PDAC), and head and neck squamous cell carcinoma (HNSCC). This integrin, which marks a myofibroblastic CAF (myCAF) subset, was found to regulate collagen matrix stiffness, contraction, and fibroblast-mediated invasion, all features that are key to ECM remodeling [153, 154]. Inhibition of α11β1 with mAb 203E1, a function blocking antibody, was found to significantly impair fibroblast adhesion to collagen I, reducing CAF mediated matrix remodeling, and decreased invasion rates in PDAC myCAF in 3D cultures. These findings emphasize the potential of integrin α11β1 as a target for disrupting tumor-stroma crosstalk and barrier formation in solid tumors [154].

Although few clinical agents have been developed to specifically target ECM-remodeling integrins, preclinical studies support the notion that disrupting integrin-MMP cooperation, or integrin-stromal activity, could impair tumor mediated matrix degradation and invasion [155, 156].

#### Targeting mechanotransduction

Targeting integrin-mediated mechanotransduction is as a compelling therapeutic strategy, as tumor-associated matrix stiffness and mechanical signaling critically regulate cancer progression. Matrix stiffness is a critical biomechanical regulator of cancer behavior, with recent findings implicating that mechanotransduction is involved in the maintenance tumor traits [157, 158]. In HCC, heterogenous matrix stiffness across tumor regions was shown to correlate with stem-like phenotypes in tumor cells. Mechanistically, stiff substrates activate the integrin-YAP axis without an increase in YAP expression, and instead it enhances nuclear translocation through dephosphorylation of YAP. Pharmacological inhibition of integrins (ATN-161) or YAP (verteporfin) reversed stiffness-induced stem-like features in both in vivo and in vitro, confirming that integrin-mediated mechanotransduction plays a primary role for matrix-regulated CSC traits [158].

Wang et al. demonstrated that a matrix stiffness plays a critical role in promoting tumor growth, stemness, and chemoresistance in HCC through integrin β1-FAK-ERK1/2-NF-κβ signaling. Using tunable polyacrylamide hydrogels and atomic force microscopy, they showed that stiffer substrates enhance both the tumorigenic and invasive capacity of liver cancer cells, while increasing the activation of the β1 integrin along with its downstream effectors. Inhibition of FAK, ERK, and NF-κβ signaling disrupted these pro-tumorigenic effects. Additionally, combining the β1 inhibitor, GLPG0187, with standard chemotherapy significantly reduced tumor growth and survival of HCC in xenograft models [159]. These findings highlight the importance of integrin β1 in linking matrix stiffness to the progression of HCC and treatment resistance.

#### Targeting angiogenesis

Recent work has expanded on the well-established role of integrins in orchestrating angiogenesis, this has led to more attention towards the development of integrin targeted therapies, which aim to disrupt formation of blood vessels.

Integrin β1 has been identified as a potential key mediator of angiogenesis and radioresistance in laryngeal squamous cell carcinoma (LSCC) [160]. Evidence from a recent study solidifies integrin β1's role, demonstrating that luteolin compromises the survival of LSCC by simultaneously overcoming radioresistance and inhibiting angiogenic pathways through β1 downregulation. The observed synergy between luteolin and irradiation suppressed FaDu cell proliferation, introduced apoptotic death, and impaired vascular endothelial growth factor A (VEGFA)-dependent tube formation in vitro. Importantly, the overexpression of integrin β1 drove both neovascularization and radioresistance, establishing its function as a regulator in these processes. The translational relevance of these findings was confirmed in vivo, where co-administration of luteolin and radiotherapy reduced tumor progression and vascular density, hallmarked by a downregulation of VEGFA, integrin β1, and CD31. Consequently, luteolin emerges as a multi-mechanistic agent capable of disrupting the pro-tumorigenic and therapy-resistant mechanisms in β1-expressing LSCC. [161].

Similarly, Li et al. demonstrated that HM-3-HSA, a fusion protein that is derived from the anti-tumor peptide HM-3 and human serum albumin (HSA), exerts potent anti-angiogenic and anti-tumor activity in HCC. When compared to its parent compound HM-3, HM-3-HSA showed greater pharmacokinetics, and also significantly suppressed tumor growth and neovascularization in HCC xenografts. Mechanistically, HM-3-HSA binds selectivity to the αvβ3 and α5β1 integrins on endothelial cells, thereby causing disruption of downstream pro-angiogenic signaling through inhibition of the phosphorylation of FAK, Src, and PI3K. In vitro, HM-3-HSA was found to impair key functions such as proliferation, migration, invasion and tube formation, indicating angiogenesis specificity. This anti-angiogenic activity was also observed in vivo, where tumor necrosis was observed, possibly by vascular deprivation. These findings position HM-3-HSA as a promising therapeutic option for integrin-mediated tumor angiogenesis in HCC [162].

While the therapeutic strategies described above demonstrate the potential of targeting integrin-mediated pathways in cancer, they also reveal important challenges in translating integrin biology into effective treatments. These challenges highlight key gaps between preclinical and clinical implementation, including limitations in therapeutic design and contextual applicability. These issues are discussed in the context of clinical studies in the following section.

### Failed therapies

Despite extensive preclinical validation of integrin-targeted therapies, translation to clinical studies has largely remained disappointing, with most integrin inhibitors failing to show efficacy in late-stage trials [163].

The most well-known integrin-targeting compound is cilengitide, a cyclic RGD peptide that inhibits both αvβ3 and αvβ5 integrins. Preclinical studies demonstrated promising anti-angiogenic and anti-invasive activity in glioma mouse models, alongside radio sensitization of GBM in both glioma cells and orthotopic rat glioma xenograft models [164]. Despite these promising results, cilengitide failed to improve clinical outcomes in two major trials. In the CENTRIC phase III trial, cilengitide paired with standard chemotherapy failed to improve overall survival in patients with O6-methylguanine-DNA methyltransferase (MGMT)-methylated GBM, the subgroup previously believed to benefit most from this treatment. The median overall survival of those who received cilengitide (26.3 months) was identical to those who received standard chemotherapy alone. Additionally, none of the predefined clinical subgroups showed any benefit from cilengitide [165]. Similarly, in the CORE phase II trial no meaningful dose–response relationship was observed. However modest, albeit statistically modest improvements were detected in MGMT-unmethylated models, despite prior predictions that cilengitide would be more effective in MGMT-methylated GBM [166].

Volociximab, a chimeric monoclonal antibody which targets integrin α5β1, was developed to disrupt fibronectin-integrin interactions while also inducing apoptosis in proliferating endothelial cells. In a phase II study in metastatic clear cell renal cell carcinoma, stable disease was reported in 80% of patients, however only 1 patient from 40 achieved partial response and the median time to progression was 4 months [167]. In platinum-resistant ovarian cancer, volociximab monotherapy yielded minimal benefit, with most patients progressing on treatment [168]. A combination study with pegylated liposomal doxorubicin similarly failed to improve progression-free survival, leading to the study being terminated [169]. While early-phase trial demonstrated favorable safety profile and moderate biological activity, it did not warrant advancement to phase III clinical trials. Despite this volociximab’s safety and disease stabilization suggests that targeting integrin α5β1 could warrant further investigation [170].

Finally, abituzumab, a humanized monoclonal antibody targeting integrin αv heterodimers, was designed to block integrin-ECM interactions, impair tumor cell adhesion and motility and induce apoptosis. It demonstrated significant preclinical activity [171]. However, despite evidence linking αv integrins to metastatic colonization, abituzumab failed to demonstrate efficacy in phase II trials. In the PERSEUS phase II trial in patients with metastatic castration-resistant prostate cancer, abituzumab failed to improve progression-free survival when compared to placebo, however reduced cumulative incidence of bone lesion progression was observed over 2 years [172]. Similarly, in the POSIEDON phase I/II trial in a KRAS wild-type colorectal cancer, combining abituzumab with cetuximab and irinotecan failed to improve progression-free survival when compared to standard therapy alone. While higher tumor expression of integrin αvβ6 was associated with greater overall survival, this was not explored conclusively [171]. Ultimately, abituzumab did not demonstrate sufficient efficacy for phase III trials.

Collectively, the failure of integrin-targeted therapies in clinical trials likely reflects a combination of translational and biological challenges. Integrin signaling is highly context-dependent and functionally redundant, enabling compensatory activation of alternative integrins or pathways upon single integrin inhibition. In addition, integrins can play distinct roles at different stages of tumor progression, and their inhibition may therefore be insufficient in established tumors. Finally, limitations of preclinical models in fully simulating tumor heterogeneity and microenvironmental complexity may contribute further to discrepancies between preclinical and clinical efficiencies (Table 1).

**Table 1:** Ongoing clinical trials regarding integrin targeted therapy

Integrins	Treatment	Disease	Mode of Delivery	Phase	No. of Patients	Status	Reference ID
αvβ3	177Lu-AB-3PRGD2	αvβ3-positive tumors (adenoid cystic carcinoma, intrahepatic cholangiocarcinoma, and uterine leiomyosarcoma)	Intravenous	Early Phase 1	10	Recruiting	NCT05013086
αvβ3	ProAgio	Pancreatic adenocarcinoma, gastrointestinal malignancies	Intravenous	Phase 1	58	Recruiting	NCT05085548
αvβ6	[68 Ga]Ga DOTA-5G and [177Lu]Lu DOTA-ABM-5G	Non-small cell lung cancer	Intravenous	Phase 1	40	Recruiting	NCT06228482
αvβ3	ProAgio and Gemcitabine	Triple-negative breast cancer	Intravenous	Phase 1/2	51	Not Yet Recruiting	NCT06460298
α4β7	Infliximab and Vedolizumab	Genitourinary cancer, and melanoma	Intravenous	Phase 1/2	100	Recruiting	NCT04407247
αvβ3 αvβ5	[177Lu]Lu-FF58	Pancreatic ductal adenocarcinoma, gastroesophageal adenocarcinoma, and glioblastoma multiforme	Intravenous	Phase 1	116	Recruiting	NCT05977322
β6	SGN-B6A	Non-small cell lung carcinoma, Squamous Cell Carcinoma of Head and Neck, HER2 Negative Breast Neoplasms, Esophageal Squamous Cell Carcinoma, Esophageal Adenocarcinoma, Gastroesophageal Junction Adenocarcinoma, Ovarian Neoplasms, Cutaneous Squamous Cell Cancer, Exocrine Pancreatic Adenocarcinoma, Urinary Bladder Neoplasms, Uterine Cervical Neoplasms, Stomach Neoplasms	Intravenous	Phase 1	824	Recruiting	NCT04389632
αVβ3	177Lu-TATE-RGD	αvβ3-positive tumors (specific tumors are not mentioned)	Intravenous	Early Phase 1	10	Recruiting	NCT06632873
αvβ8	CRB-601	Advanced solid tumors (specific tumors are not mentioned)	Intravenous	Phase 1/2	156	Recruiting	NCT06603844

## Conclusion

Integrins have emerged as crucial regulators in cancer biology by orchestrating key processes such as stemness, plasticity, immune evasion, ECM remodeling, dormancy, and angiogenesis. Their bidirectional signaling and diverse ligand-binding capacities enable tumor cells to engage with the TME, adapt to stress, and resist therapy. Collectively, these findings position integrins as dynamic regulators that integrate CSC-intrinsic programs with microenvironmental and extracellular cues.

This review proposes the angle that integrin signaling plays a complex and central role in CSC phenotype and therapy resistance, while also being integral to the remodeling of the ECM and formation and maintenance of the TME. Importantly, integrin function is highly context dependent, shaped by tumor state, ECM composition, mechanical cues, and stromal interactions, which together determine signaling outcomes in cancer.

Despite strong preclinical evidence, integrin-targeted therapies have encountered substantial challenges in clinical translation, with limited efficacy observed in late-stage trials. These outcomes highlight the complexity of integrin signaling in cancer and unresolved challenges in translating mechanistic insight into consistent therapeutic benefit.

However, deeper understanding of integrins and their cancer related mechanisms offers opportunities for more precise and context-aware intervention. As research advances, and the role of integrin variants in various cancers are better defined, emerging strategies that target both CSC programs and the TME may improve the therapeutic efficacy of integrin-directed approaches.

## Data Availability

No datasets were generated or analysed during the current study.

## Abbreviations

ECM: Extracellular Matrix
ICAM: Intracellular Adhesion Molecule
APC: Antigen-Presenting Cell
TME: Tumor Microenvironment
CSC: Cancer Stem Cell
RGD: Arginine-Glycine-Aspartic Acid
mAb: Monoclonal Antibody
PSI: Plexin-Sempahorin-Integrin Domain
MIDAS: Metal-Ion Dependent Adhesion Site
AMIDAS: Adjacent Site to the MIDAS
TGFβ: Transforming Growth Factor β
VCAM-1: Vascular Cell-Adhesion Molecule 1
ROS: Reactive Oxygen Species
GFOGER: Glycine-Phenylalanine-Hydroxyproline-Glycine-Glutamic Acid-Arginine
FAK: Focal Adhesion Kinase
HER-2: Human Epidermal Growth Factor Receptor 2
GBM: Glioblastoma
AML: Acute Myeloid Leukemia
CD: Cluster of Differentiation
GSCs: GBM Stem Cells
BC: Breast Cancer
SOX2-Sex: Determining Region Y-box 2
PI3K: Phosphoinositide 3-Kinase
Akt: Protein Kinase B
mTOR: Mammalian Target of Rapamycin
VEGF: Vascular Endothelial Growth Factor
NRP-1: Neuropilin-1
GSC: GBM Stem Cell
SCC: Squamous Cell Carcinoma
Ras: Rat Sarcoma
MEK: Mitogen-Activated Protein Kinase Kinase
ERK: Extracellular signal-regulated kinase
GLI1: Glioma-Associated Oncogene Homolog 1
BMI-1: B-Cell-Specific Moloney Murine Leukemia Virus Integration Site 1
TAZ: Transcriptional coactivator with PDZ-binding motif
TRIM11: Tripartite Motif-Containing Protein 11
PFK: Phosphofructokinase
HIF1α: Hypoxia-Inducible Factor 1-α
IFG1R: Insulin-Like Growth Factor-1 Receptor
EMP: Epithelial-Mesenchymal Plasticity
NSCLC: Non-Small Cell Lung Cancer
AMPK: AMP-Activated Protein Kinase
Src: Sarcoma (proto-oncogene tyrosine-protein kinase)
PGC1α: Peroxisome Proliferator-Activated Receptor-Gamma Coactivator 1α
EV: Extracellular Vesicle
YES1: Yamaguchi Sarcoma Viral Oncogene Homolog 1
YAP1: Yes-Associated Protein 1
TM: Tumor Microtube
ATR: Ataxia Telangiectasia and Rad3-Related Protein
UNC13D: Munc13-4
Rab11: Ras-Related Protein Rab-11
FA: Focal Adhesion
c-Met: Cellular-Mesenchymal Epithelial Transition Factor
FERM: Four-Point-One, Ezrin, Radixin, Moesin Domain
T_reg_: Regulatory T-cells
LAP: Latency-Associated Peptide
TGFβR1/2: TGF-β Receptor
SMAD2/3: Mothers against Decapentaplegic Homolog 2/3
NK: Natural Killer
MMP2: Matrix Metalloproteinase 2
MMP9: Matrix Metalloproteinase 9
CRISPR: Clustered Regularly Interspaced Short Palindromic Repeats
OSCC: Oral Squamous Cell Carcinoma
KRT17: Keratin 17
EGFR: Epidermal Growth Factor Receptor
miRNA: Micro-RNA
OC: Ovarian Cancer
OCSC: OC Stem Cell
ALDH: Aldehyde Dehydrogenase
ILK: Integrin-Linked Kinase
Fzd7: Frizzled-7
Ak: Adenylate Kinase
EMT: Epithelial-Mesenchymal Transition
SFK: Src Family Kinase
IL6: Interleukin-6
STAT3: Signal Transducer and Activator of Transcription 3
POSTN: Periostin
EOC: Epithelial Ovarian Cancer
STn: Sialylated Tn
TAF: Tumor Associated Fibroblast
CAF: Cancer Associated Fibroblast
Cdc42: Cell division control protein 42
Rac1: Ras-related C3 Botulinum Toxin Substrate 1
NGF: Nerve Growth Factor
Glc: Collagen-Glucose-Functionalized Collagen
CXCR4: C-X-C Chemokine Receptor 4
EpCAM: Epithelial Cell Adhesion Molecule
µPA: Urokinase Plasminogen Activator
TAM: Tumor Associated Macrophages
ROCK: Rho-Associated Kinase
α-SMA: α-Smooth Muscle Actin
KLF10: Krüppel-like Factor 10
COX2: Cyclooxygenase 2
MT1: MMP-Membrane-type 1 MMP
myCAF: Myofibroblastic CAF
PDAC: Pancreatic Ductal Adenocarcinoma
HNSCC: Head and Neck Squamous Cell Carcinoma
HCC: Hepatocellular Carcinoma
NF-κβ: Nuclear Factor κβ
TBK1: TANK-binding Kinase 1
VE-cadherin: Vascular Endothelial Cadherin
HUVEC: Human Umbilical Endothelial Vesicle
RaIB: Ras-like Protein B
KRAS: Kirsten Rat Sarcoma Viral Oncogene Homolog
PD-1: Programmed Cell Death Protein 1
GIC: Glioma-Initiating Cell
LSCC: Laryngeal Squamous Cell Carcinoma
VEGFAQ: Vascular Endothelial Growth Factor A
HSA: Human Serum Albumin
MGMT: O6-Methylguanine-DNA Methyltransferase
